# The Attitude of Great Writers Towards Disease

**Published:** 1892-12-03

**Authors:** 


### The Attitude of Great Writers Towards Disease.

### XVII.—CHARLES KINGSLEY.

"Disease is a sort of sporting with your true doctor. He blazes away at one where he sees it, as he would at a bear or a lion; the very sight of it excites his organ of destructiveness. Don't you understand me? You hate sin, you know. Well, I hate disease. Moral evil is your devil, and physical evil is mine. I hate it little or big; I hate to see a fellow sick; I hate to see a child ricketty and pale; I hate to see a speck of dirt in the street; I hate to see anything wasted, anything awry, anything going wrong; I hate to see water-power wasted, manure wasted, land wasted, muscle wasted, pluck wasted, brains wasted. I hate neglect, incapacity, idleness, ignorance, and all the disease and misery which spring out of that. There's my devil, and I can't help for the life of me going right at his throat, wheresoever I meet him!"

Here, in Tom Thurnall's words, we have Charles Kingsley's "attitude towards disease." Among all the varied strands which went to the weaving of his reputation as poet, novelist, preacher, and reformer, the most prominent of all is his devotion to the cause of sanitary science. "I am tired of most things in the world," he writes in 1859, "of sanitary reform I shall never grow tired. No one can accuse a man of being sentimental over it, or of doing too much in it. There can be no mistake about the saving of human lives, and the training up a healthy generation."

His were not at first sight the physical or mental qualities which might incline a man to this life-long struggle with filth and infection. The highly sensitive nature, keenly delighting in everthing strong, healthy, and beautiful, might have given the world its poet or preacher, but seemed little likely to develop a hard-headed practical man of drains. For "his senses were acute to an almost painful degree. The sight of suffering and the foul scent of the sick-room, well used as he was to both—would haunt him for hours." What hindered him then from turning aside from investigations and revelations which "saddened and sickened" him to congenial work and passionately-loved persuits? What but "the love of Christ constraining him"? Early in the combat he wrote, "He has made the Word of the Lord like fire within my bones, giving me no peace till I have spoken out." And again, "Did He let me become a strong, daring, sporting wild man-of-the-woods for nothing?" It was those two factors of devotion to the Master's call and of free delight in Nature for her own sake, which worked in him the enthusiasm of the reformer, vowed to win for his brethren what he valued most himself.

Unlike many reformers, he began his work at home. It was in nursing with his own hands his sick parishioners at Eversley that he came face to face with the stern truths about the spread of diseases, which he was afterwards to proclaim with such irresistible force. His wonderful power of sympathy with the "masses" took its rise in an individual knowledge of every man, woman, and child in his parish. It was in seeking to save these few souls entrusted to him that he found, as many have found before and since, an impenetrable barrier in the brutalizing environment of the body, and following this path to its logical issue, the cause of sanitation became clear to him as the cause of God. Deferring a new edition of his poems in order to write a sanitary article. he says: "A bit of sanitary reform work is a sacred duty, from which I dare no more turn away than from knocking down a murderer whom I saw killing a woman." In fact, like the hero in "Yeast," his true epic might have begun, "Smells and the Man I sing."

Perhaps the greatest obstacle against which Kingsley had to contend was the all-prevailing impression that "'tis jidgment, sir, a jidgment of God, and we can't escape His holy will, and that's the plain truth of it." Such thoughts as these had root not only in the reckless and slothful, but in reverent hearts who, like Grace Harvey, feared to be found "fighting against God." In the great cholera scenes in "Two Years Ago," Kingsley goes straight for this paralysing delusion and hits hard. "If all parsons had preached about it for the last fifteen years, if they had told people plainly that if the cholera was God's judgment at all, it was His judgment of the sin of dirt, and that the repentance which He required was to wash and be clean in literal earnest, the cholera would be impossible in England by now." And again, "Wheresoever cholera breaks out it is some one's fault; and if deaths occur some one ought to be tried for manslaughter—I had almost said murder—and transported for life. Someone? Who? That will be settled in the next generation, when men have common sense enough to make laws for the preservation of their own lives against the dirt and covetousness and idleness of a set of human hogs."

Nothing roused his indignation more than indifference to human life, even in its feeblest, least esteemed forms. All have heard the common cant over some neglected infant, "'Twould be a mercy if 'it' was taken." If sometimes in sheer weariness at the vast accumulation of transmitted misery, you are disposed to agree, let Kingsley's words bring a truer note of faith and courage. "It is a distressing thing to see children die. God gives the most beautiful and precious thing that earth can have, and we just take it and cast it away; we cast our pearls upon the dung-hill and leave them. It does not horrify or shock me to see a man dying in a good old age. But it does shock me, it does make me feel the world is indeed out of joint, to see a child die. I believe it to be a priceless boon to the child to have lived for a week or a day."

Looking back to those times and comparing this picture with that there is much to encourage. Thank God, of no quarter in London could such things be now told as Kingsley witnessed in 1849. "I was yesterday over the cholera districts of Bermondsey; and Oh God, what I saw; people having no water to drink, hundreds of them, but the water of the common sewer which stagnated full of dead fish, cats, and dogs under their windows. At the time the cholera was raging Walsh saw them throwing untold horrors into the ditch and then dipping out the water and drinking it. And mind, these are not dirty debauched Irish, but honest hard-working artizans. Oh that I had the tongue of St. James to plead for those poor fellows."

His dreams are not all fulfilled. Not yet are the barrens of Surrey and Berkshire fertilised with the London sewage. Not yet is the water supply in the hands of the local government; not yet is a continuous supply of pure water within reach of all; and the number of public baths and lavatories is still incomplete. Fever still seizes its numerous victims; and Nature "kills and kills and kills, and is never tired of killing till she has taught man the terrible lesson he is so slow to learn, that she is only conquered by obeying her."

Yet that lesson men are getting slowly by heart at last, and as they strive each year with better heart and better methods to teach and practise it, it can never be forgotten that at a time when the laws of health were almost universally ignored, it could be said of Charles Kingsley, "He led the way."

